# Characterization of the complete chloroplast genome of *Clematis orientalis* (Ranunculaceae)

**DOI:** 10.1080/23802359.2022.2127339

**Published:** 2022-10-06

**Authors:** Yi Cui, Lihua Yang, Bingru Ma, Shaoping Ling, Jingwen Wang, Zhongming Han, Yunhe Wang

**Affiliations:** a College of Chinese Medicinal Materials, Jilin Agricultural University, Changchun, PR China; b State Key Laboratory of JLP-MOST for Ecological Restoration and Ecosystem Management, Changchun, PR China; c College of Life Science, Changchun Sci-Tech University, Changchun, PR China

**Keywords:** *Clematis orientalis*, chloroplast genome, phylogenetic tree, Ranunculaceae

## Abstract

The medicinal plant *Clematis orientalis* L. belongs to the family Ranunculaceae. In this study, we determined the complete chloroplast genome sequence of *C. orientalis* and its phylogenetic relationships with other species. The complete chloroplast genome of *C. glauca* is 159,518 bp in length, circular in structure, and has four regions including a large single-copy (LSC) region of 79,453 bp; a small single-copy (SSC) region of 17,997 bp; and two inverted repeat (IR) regions of 31,034 bp. The GC content of the genome is 38%, and those of LSC, SSC, and IR regions are 36.2, 31.4, and 42%, respectively. The genome encodes 129 unique genes, including 85 protein-coding genes, 36 *tRNA* genes, and eight *rRNA* genes. Phylogenomic analysis reveals that *C. orientalis* is most closely related to *C. aethusifolia*. This study contributes to better understanding of phylogenetic relationships of Ranunculaceae.

The plants of the genus *Clematis* belong to the family Ranunculaceae, comprise approximately 355 species worldwide (Khatere et al. 2010; Yang et al. 2019), have highly variable morphology (Yang et al. 2020) and are found mainly in China, Japan, and Korea (Wang and Li 2005; Yang et al. 2019; Chen et al. 2021). *Clematis orientalis* L. (*C. orientalis*, 1753) is a perennial plant with proven many effects in the treatment of diseases. For example, the root extract has the effect of dilating blood vessels and lowering blood pressure, the leaves are mixed with resin and placed on the wound to promote healing, the stem is used as a treatment for syphilis and pimple, dried flowers, honey, and desi ghee work together to treat rheumatism (Ishtiaq et al. 2012; Abbas et al. 2014; Hasan et al. 2019). However, little is known about the genomics of *C. orientalis*. We characterized the complete chloroplast genome of *C. orientalis* based on Illumina pair-end sequencing data analysis, which may be helpful for understanding the bioinformatics and evolution of this species, as well as assisting in phylogenetic analysis and molecular breeding.

The fresh leaves of *C. orientalis* were collected from their natural habitat in Kekedala City, Xinjiang, China (43°5254″ N, 81°0537″ E). The voucher specimen was deposited in the Herbarium of College of Chinese Medicinal Materials, Jilin Agricultural University (https://zhongyao.jlau.edu.cn; Zeliang Lü, lvzeliang@foxmail. com) under the voucher number *Y. Cui 2021011*. Genomic DNA was extracted using a QIAquick Gel Extraction kit (Qiagen, Germany), libraries were constructed following PCR amplification, and sequencing was done using an Illumina HiSeq 2500 instrument (Illumina, San Diego, CA). The sequences were assembled using metaSPAdes (Nurk et al. 2017) and gene annotation was conducted using CPGAVAS2 (Shi et al. 2019). The complete chloroplast genome sequence and annotations for *C. orientalis* were submitted to GenBank under the accession number OL333100.

The length of the chloroplast genome was 159,518 bp, and the entire genome comprised four structural domains: a large single-copy (LSC) region of 79,453 bp, a small single-copy (SSC) region of 17,997 bp, and two inverted repeat (IR) regions of 31,034 bp in size. The GC contents of the LSC, SSC, and IR regions were 36.2, 31.4, and 42%, respectively. The chloroplast genome contained 129 genes, including 85 protein-coding genes, 36 *tRNA* genes, and eight *rRNA* genes.

To confirm the phylogenetic position of *C. orientalis*, 20 complete chloroplast genome sequences of different species were downloaded from GenBank, and two species of the *Naravelia* genus in the Ranunculaceae family (*Naravelia pilulifera* and *Naravelia zeylanica*) were used as outgroups. The sequences were aligned using MAFFT (Katoh and Standley 2013), and a phylogenetic tree was constructed using maximum likelihood (ML) method by MEGA X (Kumar et al. 2018) under the GTR + I + G nucleotide substitution model with 1000 replicates. The phylogenetic position of *C. orientalis* is shown in Figure 1. *C. orientalis* was found to be closest to *C. aethusifolia* with 100% bootstrap support. The newly characterized complete chloroplast genome of *C. glauca* will provide essential data for further studies on the phylogeny and evolution of *Clematis* L., and contribute important information for understanding the physiology and evolution of Ranunculaceae.

**Figure 1.**
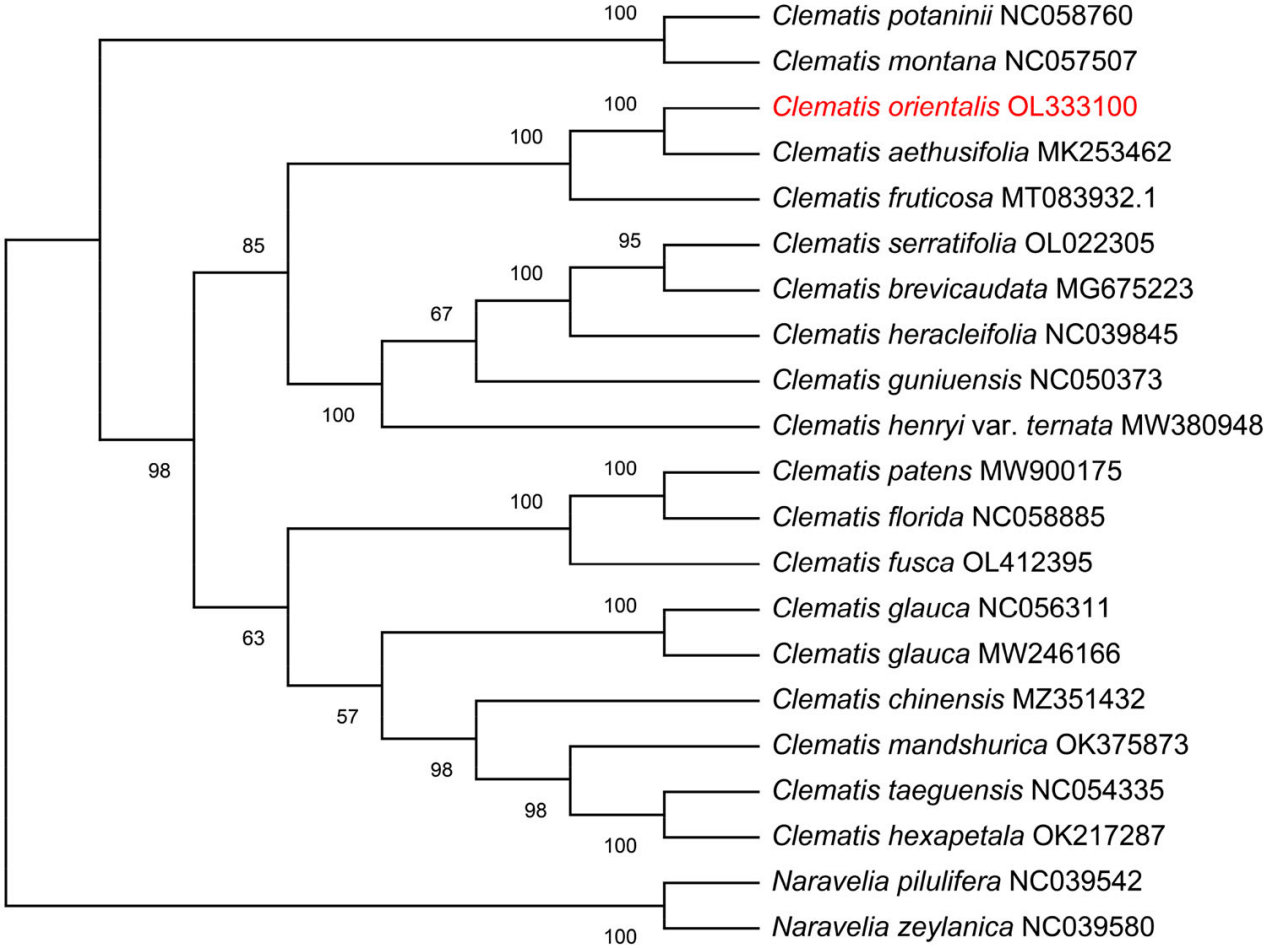
A phylogenetic tree constructed using the maximum likelihood method showing the position of *C. orientalis*. Numbers on each node show bootstrap support values from 1000 replicates.

## Ethics statement

This article does not contain any studies with human participants or animals performed by any of the authors. In this experiment, we did not collect any samples of human and animals. The specie used in this article is not endangered, protected, or personally owned.

## Data Availability

The genome sequence data that support the findings of this study are openly available in GenBank of NCBI (https://www.ncbi.nlm.nih.gov/) under the accession numbers OL333100. The associated BioProject, SRA, and Bio-Sample numbers are PRJNA764578, SRR16609052, and SAMN22567490, respectively.
